# Altered performance monitoring in Tourette Syndrome: an MEG investigation

**DOI:** 10.1038/s41598-022-12156-x

**Published:** 2022-05-18

**Authors:** Jacqueline Metzlaff, Jennifer Finis, Alexander Münchau, Kirsten Müller-Vahl, Alfons Schnitzler, Christian Bellebaum, Katja Biermann-Ruben, Valentina Niccolai

**Affiliations:** 1 Institute of Clinical Neuroscience and Medical Psychology, Medical Faculty, Heinrich-Heine-University, 40225 Duesseldorf, Germany; 2 Institute of Systems Motor Science, University of Luebeck, Luebeck, Germany; 3 Clinic of Psychiatry, Socialpsychiatry and Psychotherapy, Hannover Medical School, Hanover, Germany; 4 Department of Biological Psychology, Institute of Experimental Psychology, Heinrich-Heine-University, Duesseldorf, Germany

**Keywords:** Neuroscience, Cognitive neuroscience, Motor control, Psychiatric disorders

## Abstract

The error-related negativity (ERN) is an event-related potential component indexing processes of performance monitoring during simple stimulus-response tasks: the ERN is typically enhanced for error processing and conflicting response representations. Investigations in healthy participants and different patient groups have linked the ERN to the dopamine system and to prefrontal information processing. As in patients with Tourette Syndrome (TS) both dopamine release and prefrontal information processing are impaired, we hypothesized that performance monitoring would be altered, which was investigated with magnetencephalography (MEG). We examined performance monitoring in TS patients by assessing the magnetic equivalent of the ERN (mERN). The mERN was investigated in tic-free trials of eight adult, unmedicated TS patients without clinically significant comorbidity and ten matched healthy controls while performing a Go/NoGo task in selected frontocentral channels. The analysis of the response-related amplitudes of the event-related magnetic field showed that TS patients, in contrast to controls, did not show earlier amplitude modulation (between 70 and 105 ms after response onset) depending on response type (errors or correct responses). In both groups significant mERN amplitudes in the time-window between 105 and 160 ms after response onset were detected thus pointing at only later error processing in TS patients. In TS patients, early error-related processing might be affected by an enhanced motor control triggered by a conflict between the targeted high task performance and tic suppression. TS patients seem to tend to initially process all responses as erroneous responses.

## Introduction

A well-studied brain correlate of error processing is typically found in an event-related potential (ERP) component emerging directly after an error response. This component is called *error-related negativity* (ERN;^1^), or error negativity (Ne;^2^) and has been defined as a negative deflection with a peak between 50 and 100 ms after error response onset. It is strongest over fronto-central cortical regions^3^ and has its origin in anterior cingulate cortex (ACC)^4^ and pre-supplementary motor area (pre-SMA)^5^. The ERN has been found during performance monitoring^6^, for example in stimulus-response tasks like the Go/NoGo task, in which presented stimuli require either an overt response or a response inhibition, namely when a false response occurs in the NoGo condition^7^.

Apart from error processing as such the ERN has been suggested to monitor cognitive conflicts that occur due to two concurrently pursued responses, the correct and the erroneous one^8^: an increased top-down motor control was found to deal with risen conflicts. In line with this notion, a negativity for correct responses, the correct-response negativity (CRN), occurs in case of high response conflict in the same time window as the ERN^9^. For example, motor conflicts were shown to be accompanied by an increased CRN^10^. This was associated to a more conservative processing of each response^11^ in order to deal with risen conflicts like during response competition^4^.

A magnetic equivalent of the ERN (mERN) has been described in magnetoencephalography (MEG) studies. With a typical latency of up to 160 ms after response onset it occurs later than the ERN^12,13^. The results are inconsistent, however, with respect to the exact time-window in which it occurs^14^ and the involved cortical areas: source localisation analyses pointed at the cingulate^15^ and the frontocentral cortex^16^. Functionally, the ERN and mERN appear to be equivalent. Both are often estimated as difference between committed errors and correct responses^17^. Fitting with the conflict monitoring theory of the ERN^8^ the mERN equivalently seems to mark attention-necessity that involves increased top-down motor control^16^. One important difference in the appearance of mERN and ERN is that the equivalence of the negativity in the EEG signal is an increased positivity in the MEG signal (assessed in femtoTesla, fT).

The importance of the ERN as a helpful marker for several psychopathologies^18^ is highlighted by acknowledging the relationship between the ERN and the striatum. On the one hand, ERN has been linked to the dopamine system^19^. On the other hand, the striatum receives dopaminergic projection and is connected to the ACC^20^. For instance, reduced ERN amplitude was found in patients with schizophrenia, Parkinson’s disease^3^, X-linked dystonia parkinsonism^21^, or Huntington’s disease^22^, which was related to the dopaminergic deficit in striatal areas. Moreover, the ERN amplitude was shown to be enhanced in diseases with decreased prefrontal inhibition like obsessive compulsive disorder (OCD)^23^, attention deficit hyperactivity disorder (ADHD)^24^, and anxiety^25^. Besides, anxiety patients were also found to show an enhanced CRN^26^. Enhanced CRN amplitudes in patients with frontal brain lesions have been discussed in terms of a dysregulation of motor control^6^.

The Tourette syndrome (TS) is a neurodevelopmental disorder arising in childhood and adolescence characterised by multiple vocal and motor tics present for at least one year^27^. Tics are frequently preceded by premonitory urges, which are uncomfortable feelings or sensory phenomena accompanied by a sensation of inner pressure^28^. Increased subcortical gating of somatosensory-motor information during voluntary movement control was reported to enhance tic control in TS patients^29^. Furthermore, patients show increased self-regulatory mechanisms^30,31^ that allow them to deliberately suppress tics: dysfunctional dopamine neurotransmission modulating the activity of the basal ganglia and of the cortico-striato-thalamo-cortical (CSTC) circuit seem to influence frontal regions in TS patients^32^. Specifically, low dopamine release increases frontal cortex activation and enables TS patients to enhance their motor control and to reduce tics^33^. Frontal brain regions thus appear to play a major role and seem to contribute to increased executive control in TS.

Interestingly, brain processes subtending successful tic suppression and those related to error processing show an analogy. The ACC and the pre-SMA are activated both during tic suppression^34^ and after committing an error in choice-reaction tasks^4,5^. Also, TS patients show increased activity in the SMA after errors during a Stop-Signal task in comparison to healthy controls^35^. Notably, executive control mechanisms are also needed to avoid errors and to resolve conflicts as indicated by ERN amplitude modulation^8^. Therefore, investigating how TS patients process task-related errors and correct responses may shed light on the impact of dysfunctional executive control mechanisms in TS.

Few studies have investigated as yet the relationship between error processing and tic-related inhibitory control mechanisms in TS patients. Increased, possibly compensatory performance monitoring in TS patients was proposed to explain the behavioural finding of similar error rates and reaction times compared to healthy controls in stimulus–response tasks^29^. The proposed compensatory mechanisms, however, do not seem to be reflected in the ERN amplitude, which was found to be similar between unmedicated TS patients with no comorbidity and healthy controls^36^. Instead, comorbidity with OCD and ADHD disorders or the use of neuroleptics seem to influence TS patients’ cognitive control and error monitoring^37^ which may have contributed to higher^38,39^ and smaller ERN amplitudes^24^ in TS patients. However, a recent investigation also found an increased ERN in TS patients without any comorbidities^40^.

The aim of the current study is to contribute to the examination of a possibly altered performance monitoring system in TS by addressing error processing during a Go/NoGo task. To do so, we used MEG to examine for the first time the mERN in TS patients and matched healthy controls. The mERN was defined as the MEG response to performance errors and not as the difference wave signal. Previous studies on error processing in TS patients using EEG have been inconclusive, and as MEG is sensitive to different neuron populations compared to EEG we expected MEG to be more sensitive to subtle processing differences between TS patients and healthy controls. Nevertheless, based on the findings reported above, unmedicated TS patients without comorbidities may exhibit an mERN component similar to healthy controls when performing an erroneous response. Possibly, however, due to tic-related motor suppression together with task-related commitment to respond correctly, we expected a compensatory increase in motor conflict in TS patients reflected by the comparison of the mERN versus the magnetic CRN (mCRN) in fronto-central channels. A similarly pronounced activity after both response types (errors and correct responses), hence an additionally increased mCRN would underline the presence of an enhanced motor control for TS patients’ responses in general. A higher difference between mERN and mCRN in TS patients than in controls would, on the other hand, indicate an enhanced sensitivity for errors.

## Methods

### Participants

Twelve adult TS patients aged between 22 and 54 years (mean 37 ± 9 years, 2 female) were recruited from three specialized TS outpatient clinics (Departments of Neurology of the University Hospitals Hamburg and Düsseldorf, and Department of Psychiatry, Social psychiatry and Psychotherapy of the Hannover Medical School). All patients met the diagnostic criteria of TS according to the Diagnostic and Statistical Manual of Mental Disorders (DSM-IV-TR;^41^). To assess symptom severity, the Yale Global Tic Severity Scale (YGTSS,^42^), the Modified Rush Video-based Scale for tic rating (MRVS,^43^), and the diagnostic confidence index (DCI,^44^) were used. To assess attention deficit hyperactivity disorder the short version of the Wender Utah Rating Scale was used (WURS-k,^45^). Self-injuring and obsessive–compulsive behavior were assessed by neurologists with experience in the assessment and care of TS patients. To avoid confounding variables, the present study included only untreated patients without clinically significant comorbidity. Handedness was assessed with the Edinburgh Handedness Inventory^46^ and the Annett Hand Preference Questionnaire^47^. Descriptive data and clinical scores have been reported previously^29^.

Five TS patients had never received any medication to treat tics, the remaining 7 were off medication for at least 6 months. Twelve healthy subjects (controls) were matched to TS patients with respect to gender, age, and handedness. None of the controls had a history of neurologic or psychiatric disease. All participants had normal or corrected-to-normal vision. Participants provided written informed consent prior to the MEG, and received financial compensation for their participation. The study was in accordance with the Declaration of Helsinki and was approved by the responsible Ethics Committee (Hamburg Medical Association, study number 2514).

### Material

Presentation software (Neurobehavioral Systems, Inc., Albany, CA) was used to present visual stimuli and to record behavioural responses. The stimuli were presented in the middle of a screen at a viewing distance of one meter. Participants were required to perform a Go/NoGo task including an instructive stimulus (S1) and an imperative Go/NoGo stimulus (S2), each lasting 500 ms. S1 was presented as a cross or a square signalling participants which finger (right index or middle finger) should be used to respond. S2 was presented as a yellow or blue circle indicating whether to respond (Go) or not (NoGo). The assignment of the two stimuli used for S1 and for S2 were counterbalanced across subjects.

Trials started with a fixation cross lasting between 1000 and 2000 ms, randomly varying in 250 ms steps, followed by S1. Afterwards, S2 was presented with a stimulus onset asynchrony that randomly varied between two and six seconds in steps of one second. After S2 the fixation cross appeared for one second. Consequently, trials had an average duration of seven seconds. A total of 200 Go and 100 NoGo trials were presented in pseudo-random order in five blocks of 60 trials each.

### Procedure

At the beginning of the study each participant subscribed an informed consent form, removed all metal materials, and was prepared for MEG measurement. Afterwards, subjects were comfortably seated in a magnetically shielded room, with their right hand resting on a tablet, where two photoelectric barriers measured the onset of finger lifts. Participants were instructed to lift either the index or the middle finger as fast as possible to respond, according to the instructions (see above). TS patients were asked not to try to suppress their tics. The task began with twenty practice trials, which were then followed by the experiment.

### Data recordings

To acquire information about eye-movements and blinks, four electrooculographic (EOG) electrodes were placed horizontally and vertically around the eyes. To detect movements as well as tics, bipolar electromyographic (EMG) electrodes were placed over the right musculus frontalis, musculus orbicularis oculi, and musculus orbicularis oris and were referenced to the jaw. Further two EMG electrodes over both musculi trapezii were referenced to the clavicles. Tics were primarily scanned with video recordings and cross-checked with EMG data. For this purpose, TS patients were video recorded for offline detection of tics. Neuromagnetic brain activity was measured with a 122 planar gradiometer channels MEG system (Elekta Neuromag, Helsinki, Finland). Data were then digitized at 1000 Hz with an online bandpass filter from 0.03 to 330 Hz and saved on a computer hard disk.

### Data analysis

MEG data were analysed with Matlab (MathWorks, Natick, MA, USA) and FieldTrip, a Matlab-based software toolbox for EEG and MEG data analysis^48^. The present investigation was conducted on a pre-existing MEG data set, for clinical scores please refer to^29^.

### MEG data analysis

#### Preprocessing

Data from two controls (one female) and four TS patients (all males) were excluded from further data analysis due to a large number of blinks and movement related artifacts in the selected episodes. For the analysis of the mERN, we compared the MEG signal in response to error responses to the signal following correct responses. MEG signals following both responses with the index and with the middle finger were considered together. As for TS patients, analyses were run on tic-free episodes. Trials were response-locked and the corresponding segments ranged from 1000 ms before to 1000 ms after response onset (finger lift). Trials with more than one finger movement were excluded from analysis to exclude confounding activity due to preparational processes for upcoming additional voluntary movements^6^. Therefore, only commission errors with one movement but without corrections were considered for the error response condition: hence, one simple lift of the wrong finger following a Go-stimulus and false alarms after a NoGo stimulus without any further finger movement or immediate response correction within 1000 ms after response onset. To balance error and correct trial numbers a subgroup of trials from the correct trials group was randomly selected. More precisely, for each error, a corresponding correct response was randomly selected from the same data set of the same participant. This random sampling procedure was done for the analysis of the MEG signal related to correct responses (i.e., correct response to a Go-stimulus) in order to avoid a confound due to different trial numbers between conditions and thus signal-to-noise-ratio. Specifically, for errors committed after a NoGo stimulus, tic-free correct trials with corresponding response type (same finger) and following the corresponding visual prompt type were selected to control for presence and type of movement as well as for preceding visual stimulus. For this purpose, trials were manually and blindly selected from the overall trials list consisting of numeric values, which exclusively referred to the trigger information (i.e., response finger and prompt stimulus); crucially, this dataset did not include any hint of qualitative or identifying details of the trial, thus preventing any insight of the trial content.

**Figure 1:**
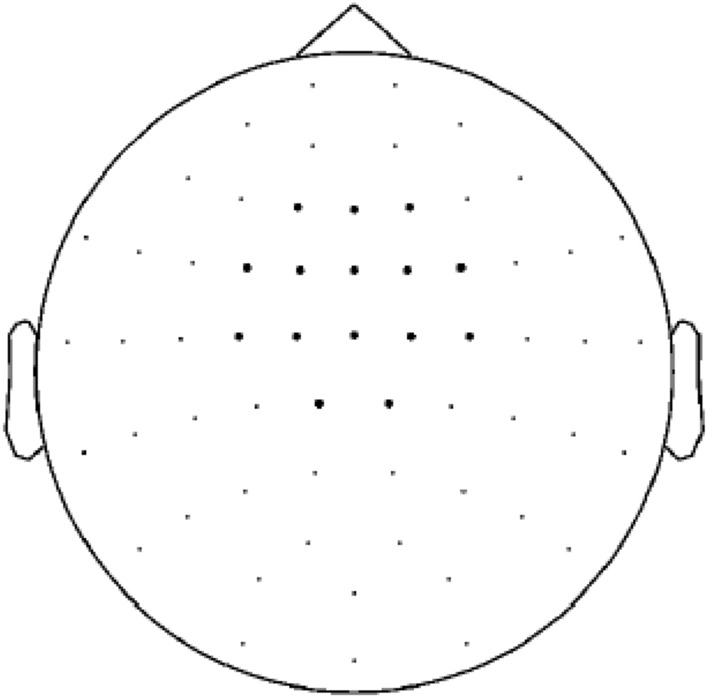
The highlighted dots represent the topographical distribution of the selected 15 fronto-central channels used for the statistical analysis of mERN amplitudes in both groups.

Muscle related artifacts and channel jumps were rejected via semi-automatic algorithms and eye related artifacts were visually detected. Due to the relatively small number of error trials, the latter artifacts were rejected only in case they influenced the selected frontocentral MEG channels (Fig. 1). On average, 6 (*SD* = 4) error trials were found for controls and 8 (*SD* = 5) for TS patients. For correct trials the numbers were the same, as error and correct trials were matched one-by-one (see above). Although these trial numbers are very low, the ERN in EEG studies has been shown to emerge even in few trials^3^, indicating that the neural response to errors is very pronounced and that the analysis does not require averaging over many trials. A low-pass filter at 40 Hz, a high-pass filter at 2 Hz, and a band stop filter at 49–51, 99–101, and 149–151 Hz were applied. Faulty channels with continuous bad signal (e.g., jumps) were replaced by means of interpolation of neighbouring channels.

#### Time-locked analysis

Trials were averaged for each subject and condition (error and correct) and horizontal and vertical planar gradients were combined. The 100 ms preceding response onset were used for baseline correction^12,13,16^. Importantly, no significant difference between the errors and the correct responses condition emerged in the baseline time-window according to a cluster-based permutation test for MEG data (*p* > 0.05; see statistics section below).

### Statistical analysis

As for behavioural data the error rates and reaction times were compared between groups by using a two-sided *t*-test. To ensure a normal distribution of behavioural data, a Kolmogorov–Smirnov test was previously conducted. To address a potential confound due to different trial numbers, a two-sided *t*-test for the comparison of the number of remaining artifact-free error rates (including false alarms and false finger movements) between groups was assessed.

According to an MEG literature-based approach^12–14^, data from two time-windows within the time range 70 to 160 ms after response onset (i.e., 70 to 105 ms and 105 to 160 ms) entered the analysis; data were averaged across 15 selected frontocentral channels (Fig. 1). Considering the multidimensionality of MEG data, a procedure that effectively corrects for multiple comparisons, the cluster-based nonparametric permutation test, was used^49^. As the focus was on possible differences in error versus correct trial-related processing between groups, interactions between response type (errors versus correct responses) and group (TS patients, controls) were analysed for each time-window first. This was done by analysing between-group differences in the error-correct difference wave. Secondly, post-hoc analyses comparing the signal for the different response types within each group and for groups within one response type were conducted using dependent and an independent *t*-tests, respectively. For all analyses, *t*-values of the time samples passing a defined alpha threshold (*p* < 0.05) were selected and clustered with spatially adjacent bins. A cluster-level statistic was then calculated by taking the sum of the *t*-values of the samples within every cluster. Nonparametric permutation testing, which consisted of computing 1000 random sets of permutations between two conditions, was used to obtain a distribution of cluster statistics and the significance level of the observed cluster (*p* < 0.05 for monodirectional hypothesis in the post-hoc comparison of response types within groups and *p* < 0.025 for bidirectional hypothesis for the comparison of the difference waveform between groups).

### Ethics approval

The study was in accord with the Declaration of Helsinki and was approved by the responsible ethics Committee (Hamburg Medical Association, study number 2514).

### Consent to participate

Participants provided written informed consent prior to the magnetoencephalography.

## Results

The Kolmogorov–Smirnov test revealed normal distributions for reaction times (for controls *p* = 0.170 and TS patients *p* = 0.200) and error rates (for controls *p* = 0.165 and TS patients *p* = 0.200). As previously shown^29^, controls and TS patients did not differ in RTs *t*(22) = 0.116, *p* = 0.909: controls responded on average after 483 ms (*SD* = 98 ms) and TS patients after 478 ms (*SD* = 101 ms). TS patients acted out their tics for several times (*M* = 46, *SD* = 20). TS patients showed a similar error rate (5%) as controls (4%) including all trials before exclusion due to artifacts with *t*(22) = 2.074, *p* = 0.244. Similar error rates relative to the remaining artifact-free trials between TS patients (2.2%) and controls (2.8%) with *t*(16) = 0.606, *p* = 0.553 suggest that the number of errors does not constitute a confound in the comparison of MEG amplitudes between patients and controls.

**Figure 2:**
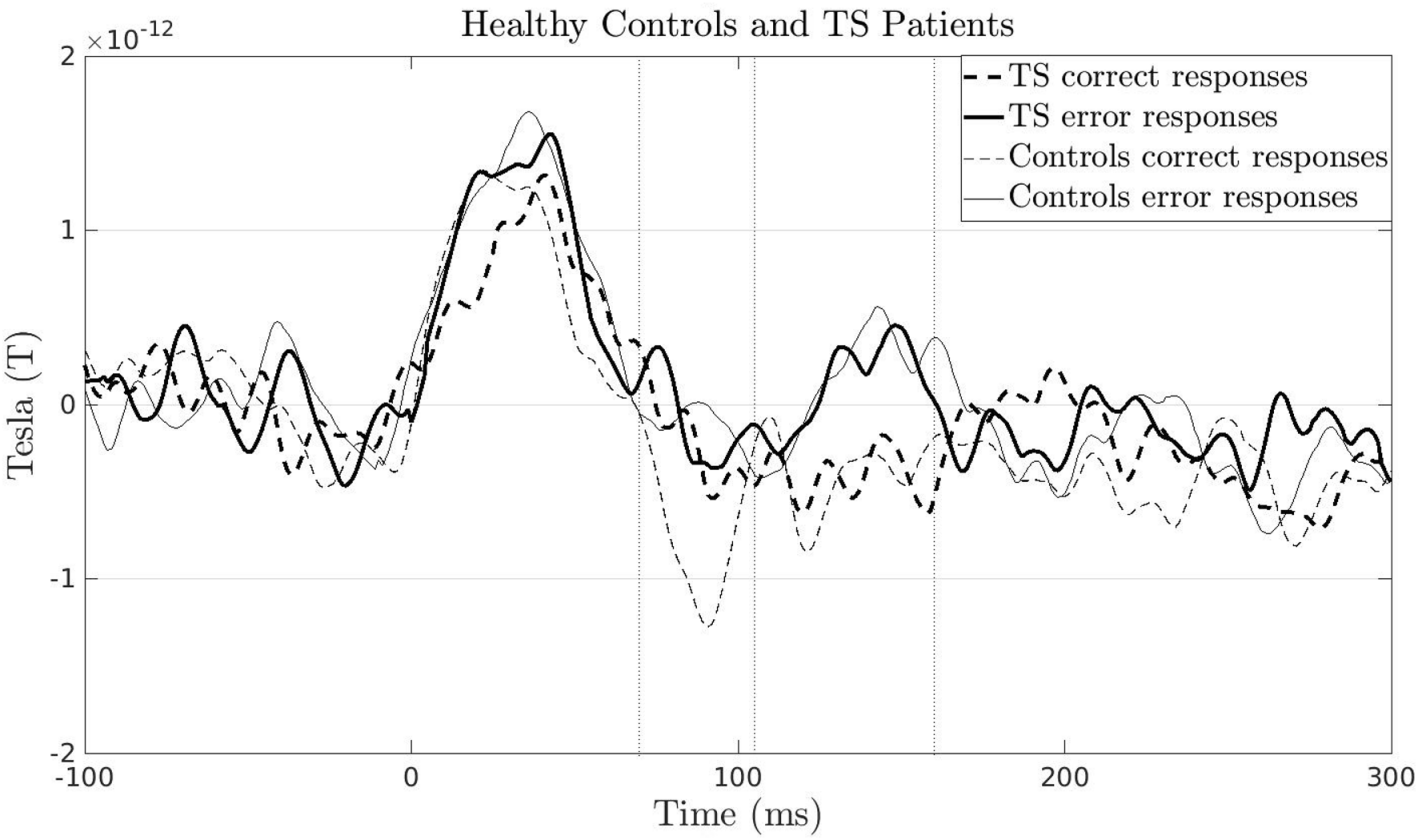
Grand averages of the amplitude of error trials (continuous lines) and correct trials (dashed lines) in healthy controls (thin lines) and TS patients (bold lines) across the selected fronto-central channels. The vertical dotted lines indicate the time range between 70 and 160 ms after response onset showing two components that were inspected separately. Confidence intervals are not provided in the figure, as this would have compromised the visibility of the MEG signals in the different conditions.

A significant interaction of the MEG amplitude between response type and group emerged in the time-window 70 to 105 ms after response onset (*p* = 0.015; *d* = 0.59; *CI* = 0.009). This finding thus revealed that the amplitude difference between error and correct trials differed between TS patients and controls in this earlier time window. Post-hoc analyses for each group separately showed a significant effect in controls (*p* = 0.003; *d* = 0.84; *CI* = 0.003; i.e. an amplitude difference between errors and correct responses), which indicated the presence of an early mERN component, but not in TS patients (no cluster emerged, no *p*-value available). In particular, the mCRN amplitude in TS patients differed significantly from that of controls (*p* = 0.019; *d* = −1.14; *CI* = 0.008). Hence, in the early time window TS patients processed their correct responses similarly to their errors. No significant interaction emerged in the second time-window (105–160 ms): here, both controls and TS patients showed significant mERN amplitudes (*p* = 0.027; *d* = 0.62; *CI* = 0.009; and *p* = 0.029, *d* = 1.41; *CI* = 0.010 respectively for the comparison of error and correct trials) (Fig. 2; Fig. S1-S3).

## Discussion

Consistent with previous research^36^, TS patients and controls showed similar behavioural performance, that is, error rates and reaction times. However, other studies reported faster reaction time for TS patients^30^. The main aim of the present study was, however, to target possible differences in the neurophysiological signature of error-related processing between TS patients and controls by means of MEG. We found that in the earlier time-window TS patients did not show an enlarged mERN in the sense of an amplitude difference between error and correct trials, while controls did. The reason for this was that in TS patients the mCRN amplitude was more pronounced compared to that of controls.

This similar processing of correct and error responses shown by TS patients may be explained in the framework of the conflict monitoring theory^4,8^. Motor conflicts were shown to be accompanied by an increased CRN^10^. Possibly, competing task demands might have resulted in the pattern of activation observed in TS patients. Successful tic-related motor suppression together with the task-related commitment to respond correctly may have resulted in a motor conflict. As shown before, such a conflict may induce increased regulation of executive control mechanisms in TS patients during task performance^30^ and affect error-related processing. Interestingly, patients with frontal brain lesions were also shown to have similar ERP amplitudes following both correct responses and errors; this was attributed to a lack of motor control^6^. In contrast, increased self-regulatory mechanisms in TS patients^30,31^ are interpreted in the light of hyperactive frontal regions. As previously shown^33^, decreased dopamine neurotransmission has been found to enhance motor control in TS patients. Therefore, both decreased and increased activity of frontal regions might influence early error processing.

The observation of a larger event-related field amplitude for errors than correct trials in the later time-window (105 to 160 ms) is in line with results from previous MEG studies with healthy subjects^14,16^, despite differences in the experimental paradigm^12^. Also, in TS patients, such a pattern has been found in a sample with a different comorbidity state^38^. TS patients and controls showed similar mERN amplitudes in this later time window, resembling previous negative findings related to ERN amplitude differences between TS patients and controls^24^^,^^36^. This suggests a later but qualitatively and timely adequate differentiation between the representation of committed errors and of correct responses^13^ in TS patients.

An alternative explanation of the present findings may point to an initial tendency of TS patients to process all their responses as erroneous responses, a hypothesis that was proposed to be reflected by the CRN^11^. At a subsequent time point they seem to differentiate between committed errors and correct responses similarly to controls. The two (sub)components may thus be linked to two different functions involved in error-related processing. Previous research^10^ indeed described two different processes that were originally related to the ERN and the following error positivity (Pe) occurring between 200 and 400 ms after response onset. A so-called comparison process of both response representations and an error detection process. The present results may suggest an impaired comparison process in TS patients due to the increased motor conflict. Similar processing of errors and correct responses was also shown in anxiety patients, assuming an ‘overactive response monitoring in general’^26^. As TS and anxiety are highly comorbid, a potential parallel could be in the conservative way of monitoring all actions as possibly erroneous. Alternatively, the early comparison process may be delayed^10^.

For subsequent investigations it would be interesting to address the subjective self-evaluation concerning response accuracy by asking the participants to evaluate their responses as erroneous or correct^15^. Objectively correct responses that are processed as an error as well as the reverse situation might affect the mERN differently^3,11^. A real-time urge intensity score like the Premonitory Urge for Tic Disorders Scale (PUTS)^28^ may also be helpful to estimate the conflict magnitude in TS patients while performing the task. Interestingly, after having committed an error and directly before the generation of tics similar brain regions are reported to be activated in TS patients^5,34^: this raises the question whether tics are processed similarly to erroneous responses. Considering that previous investigations^38^ did find the presence of ERN in TS patients with comorbid ADHD and OCD, it would be interesting in future research to tackle the motor and the attentional origin of the proposed adaptation processes. Another interesting point would be to measure controls and TS patients in a combined EEG–MEG design to better determine possible differences emerging from the analysis of the (m)ERN with the different methods^13–15^.

A limitation of the study is the small amount of errors that could be considered in the analysis^3,50^. If the tested sample size is too small, statistical effects might be overestimated or statistical power could be reduced and real effects might not be found^3^. The fact that some of the participants had to be excluded from analysis reduces the chance of finding real effects or underlines that the found effects display real differences in mERN between groups^50^. Our results show, however, that despite the small number of trials and the small sample, the mERN was comparable to previous studies. In fact, the ERN is a strong component that can be detected also in a small amount of trials, even with only six trials per participant^7,51^. However, due to the small sample size, the comparable difference wave amplitudes in the late time window between patients and controls have to be interpreted carefully. In ERN analyses including group comparisons a small number of errors may decrease statistical power^50^. Finally, considering that the applied manual and blind random selection of a subset of correct trials before preprocessing hinders the replicability of the current results, it would be important for future studies to aim at an enhanced robustness of findings by processing multiple subsets of correct response trials and inspecting results consistency.

All in all, the present findings may suggest a different early, but similar later error-related processing in TS patients in comparison to controls. One reason may be that due to concurrent tic suppression there is increased conflict in TS patients, which leads to an enhanced mCRN in the early time window. Alternatively, TS patients possibly first process their correct responses as erroneous responses to reach a normal behavioural task performance. TS patients’ enhanced executive control seems to enable them to reach high performance. Future studies should replicate the findings in a lager sample and address which of the two explanations for our result pattern is more likely.

## Supplementary Information

Supplementary Information.

## Data Availability

The present investigation was conducted on a pre-existing MEG data set (please refer to Biermann-Ruben et al.^29^). Due to the different data security standards of 2009 data is not open accessible but can be fully requested by addressing the corresponding author. The script including analysis steps is publicly available in the OSF repository (project: ERF Tourette Syndrome; https://osf.io/7tvge/files/).
