# Supplementary material for: Altered performance monitoring in Tourette Syndrome: an MEG investigation
Source: Sci Rep. 2022 May 18;12:8300. doi: 10.1038/s41598-022-12156-x (PMC9117680; doi:10.1038/s41598-022-12156-x)
Supplement: Supplementary file 1 — Supplementary Information. [file 41598_2022_12156_MOESM1_ESM.docx]

Supplementary Material


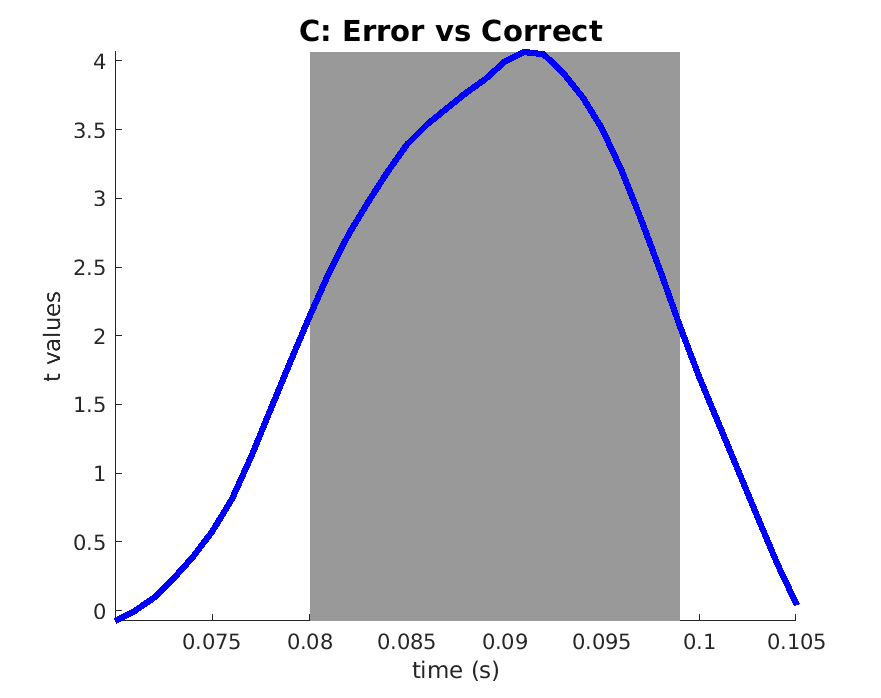


**Figure S1.** T-values for the contrast error versus correct response condition in healthy controls, averaged over the selected fronto-central channels across the time window between 70 and 105 ms after response onset; the grey square indicates significant t-values.


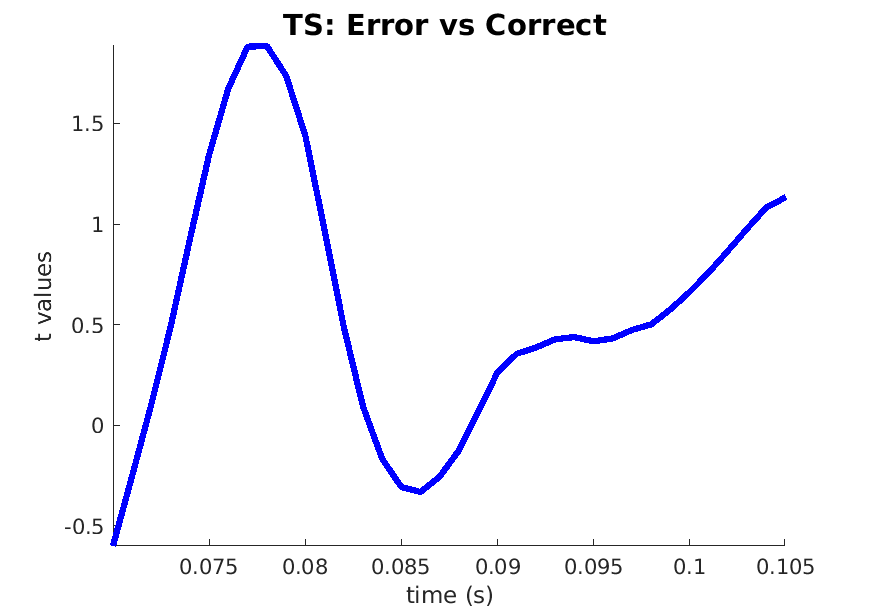


**Figure S2.** T-values for the contrast error versus correct response condition in TS patients, averaged over the selected fronto-central channels across the time window between 70 and 105 ms after response onset; no significant t-values.


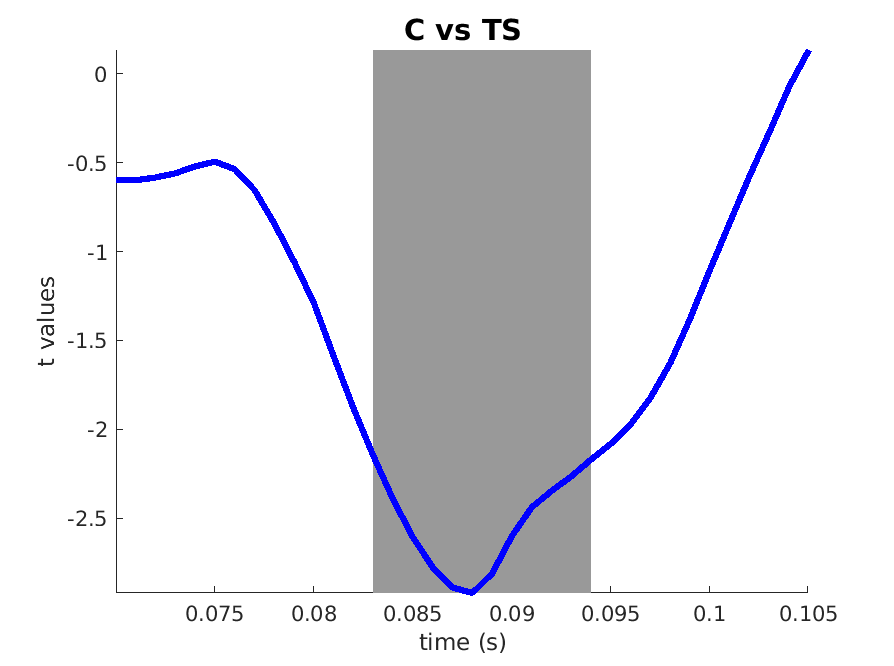


**Figure S3.** T-values for the contrast of the mCRN between groups, averaged over the selected fronto-central channels across the time window between 70 and 105 ms after response onset; the grey square indicates significant t-values.

Financial Disclosures: Jacqueline Metzlaff: She did not receive any funding, there is no conflict of interest. Jennifer Finis: She also did not receive any funding, there is no conflict of interest. Kirsten Müller-Vahl has received financial or material research support from EU (FP7-HEALTH-2011 No. 278367, FP7-PEOPLE-2012-ITN No. 316978) DFG: GZ MU 1527/3-1 and GZ MU 1527/3-2, BMBF: 01KG1421, National Institute of Mental Health (NIMH), Tourette Gesellschaft Deutschland e.V. Else-Kröner-Fresenius-Stiftung, GW pharmaceuticals, Almirall Hermal GmbH, Abide Therapeutics, and Therapix Biosiences. She has received consultant's honoraria from Abide Therapeutics, Boehringer Ingelheim International GmbH, Bionorica Ethics GmbH, CannaMedical Pharma GmbH, Canopy Grouth, Columbia Care, CTC Communications Corp., Demecan, Ethypharm GmbH, Eurox Deutschland GmbH, Global Praxis Group Limited, MCI Germany, Lundbeck, Sanity Group, Stadapharm GmbH, Synendos Therapeutics AG, and Tilray. She is an advisory/scientific board member for CannaMedical Pharma GmbH, Bionorica Ethics GmbH, CannaXan GmbH, Canopy Growth, Columbia Care, Ethypharm GmbH, IMC Germany, Leafly Deutschland GmbH, Sanity Group, Stadapharm GmbH, Synendos Therapeutics AG, Syqe Medical Ltd., Therapix Biosciences Ltd., Tilray, von Mende Marketing GmbH and Wayland Group. She has received speaker’s fees from Aphria Deutschland GmbH, Almirall, Cogitando GmbH, Emalex, Eurox Deutschland GmbH, Ever pharma GmbH, Meinhardt Congress GmbH, PR Berater, Spectrum Therapeutics GmbH, Takeda GmbH, Tilray, Wayland Group. She has received royalties from Deutsches Ärzteblatt, Der Neurologie und Psychiater, Elsevier, Medizinisch Wissenschaftliche Verlagsgesellschaft Berlin, and Kohlhammer. She served as a guest editor for Frontiers in Neurology on the research topic “The neurobiology and genetics of Gilles de la Tourette syndrome: new avenues through large-scale collaborative projects”, is an associate editor for “Cannabis and Cannabinoid Research” and an Editorial Board Member of “Medical Cannabis and Cannabinoids” und “MDPI-Reports” and a Scientific board member for “Zeitschrift für Allgemeinmedizin”. Alexander Münchau has been a member of the advisory board of the Tourette Gesellschaft Deutschland (German Tourette syndrome association); speaker of the Lübeck Center for Rare Diseases; received honoraria for lectures: Pharm Allergan, Ipsen, Merz Pharmaceuticals, Actelion, GlaxoSmithKline, Desitin, Teva, Takeda; consultancies: Desitin, Merz Pharmaceuticals, Admedicum, PTC Therapeutics; received support from foundations: Possehl-Stiftung (Lübeck, Germany), Margot und Jürgen Wessel Stiftung (Lübeck, Germany), Tourette Syndrome Association (Germany), Interessenverband Tourette Syndrom (Germany), CHDI, Damp-Stiftung (Kiel, Germany); received academic research support from Deutsche Forschungsgemeinschaft (DFG): projects 1692/3-1, 4-1, SFB 936, FOR 2698 (project numbers 396914663, 396577296, 396474989); European Reference Network – Rare Neurological Diseases (ERN – RND; Project ID No 739510); received royalties for the book Neurogenetics (Oxford University Press); received Advisory Boards from German Tourette syndrome Association; Alliance of patients with chronic rare diseases. Alfons Schnitzler is an employee at Heinrich-Heine-University and received through his institution funding for his research from the German Research Council, the German Ministery of Education and Health, and the Helmholtz Association. A.S. has been serving as a consultant for Medtronic Inc, Boston Scientific, and St. Jude Medical and has received lecture fees from Boston Scientific, St. Jude Medical, Medtronic GmbH, UCB, MEDA Pharma, Teva Pharma, and GlaxoSmithKline. Christian Bellebaum is emploved at the Heinrich-Heine-University. Katja Biermann-Ruben is employed at the Heinrich-Heine-University. Valentina Niccolai: SFB 991/B03.
